# PAX8 activates metabolic genes via enhancer elements in Renal Cell Carcinoma

**DOI:** 10.1038/s41467-019-11672-1

**Published:** 2019-08-20

**Authors:** Melusine Bleu, Swann Gaulis, Rui Lopes, Kathleen Sprouffske, Verena Apfel, Sjoerd Holwerda, Marco Pregnolato, Umut Yildiz, Valentina Cordoʹ, Antonella F. M. Dost, Judith Knehr, Walter Carbone, Felix Lohmann, Charles Y. Lin, James E. Bradner, Audrey Kauffmann, Luca Tordella, Guglielmo Roma, Giorgio G. Galli

**Affiliations:** 1 Disease Area Oncology, Novartis Institute for Biomedical Research, Basel, Switzerland; 2 Chemical Biology & Therapeutics, Novartis Institutes for Biomedical Research, Basel, Switzerland; 3 Department of Molecular and Human Genetics, Baylor College of Medicine, Houston, TX USA; 4 Novartis Institutes for Biomedical Research, Cambridge, MA USA; 5 Present Address: Friedrich Miescher Institute for Biomedical Research and University of Basel, Basel, Switzerland; 6 Present Address: Division of Stem Cells and Cancer, German Cancer Research Center (DKFZ), Heidelberg, Germany; 7 Present Address: Prinses Maxima Center for Pediatric Oncology, Utrecht, Netherlands; 8 Present Address: Stem cell program, Children’s Hospital Boston and Department of Genetics, Harvard Medical School, Boston, USA; 9 Present Address: Autoimmunity, Transplantation and Inflammation, Novartis Institutes for BioMedical Research, Basel, Switzerland

**Keywords:** Cancer metabolism, Urological cancer, Target identification, Epigenetics

## Abstract

Transcription factor networks shape the gene expression programs responsible for normal cell identity and pathogenic state. Using Core Regulatory Circuitry analysis (CRC), we identify PAX8 as a candidate oncogene in Renal Cell Carcinoma (RCC) cells. Validation of large-scale functional genomic screens confirms that PAX8 silencing leads to decreased proliferation of RCC cell lines. Epigenomic analyses of PAX8-dependent cistrome demonstrate that PAX8 largely occupies active enhancer elements controlling genes involved in various metabolic pathways. We selected the ferroxidase Ceruloplasmin (CP) as an exemplary gene to dissect PAX8 molecular functions. PAX8 recruits histone acetylation activity at bound enhancers looping onto the CP promoter. Importantly, CP expression correlates with sensitivity to PAX8 silencing and identifies a subset of RCC cases with poor survival. Our data identifies PAX8 as a candidate oncogene in RCC and provides a potential biomarker to monitor its activity.

## Abstract (web summary)

Transcription factors are critical regulators of cell identity. Here, the authors use computational and functional genomic approaches to show an oncogenic role of PAX8 in renal cancer. Mechanistic dissection of PAX8 functions reveal its role in activating genes associated with metabolic pathways.

## Introduction

Gene expression of a given cell type is determined by a tightly regulated network of critical transcription factors (TFs) driving the expression of essential genes for cell identity^1^. TFs control their transcriptional output by engaging proximal or distal regulatory elements such as promoters and enhancers^2^. Active regulatory elements share the presence of H3K27ac marks at neighboring nucleosomes, leading to open chromatin and accessibility by TFs^2^. In the three-dimensional spatial organization, enhancer elements are able to engage gene promoters by DNA looping and trigger the RNA polymerase machinery for effective transcription^3^.

In recent years, epigenomic profiling and cataloging of active enhancers allowed the identification of networks of TFs involved in reciprocal coregulation as well as maintenance of cell-type-specific gene expression^4,5^. These transcriptional programs are essential for cell identity in normal conditions, yet they are frequently deregulated during tumorigenesis^6,7^. Importantly, cancer cells are often addicted to transcriptional networks, suggesting that the inhibition of oncogenic TFs results in the disruption of aberrant gene expression and subsequent cell differentiation or death^6^. Therefore, the development of novel drugs to target TFs promises potential for the treatment of cancer^8^.

Renal cell carcinoma accounts for 2% of all cancer cases and is responsible for >130,000 yearly deaths worldwide. While RCC is a heterogeneous disease encompassing a large number of histological subtypes, clear cell RCC (ccRCC) is the most frequent subtype (>80% of the cases) and primarily responsible for mortalities (reviewed in ref. ^9^). The most frequently mutated gene in ccRCC is the Von Hippel Lindau (VHL) gene whose genetic aberration is a truncal event in kidney tumor evolution^10^. Due to its role as an E3 ubiquitin ligase complex, loss of VHL leads to stabilization and activation of the TFs HIF1α and HIF2α that control angiogenesis, glycolysis, and apoptosis. VHL loss seems insufficient to induce ccRCC and additional second-hit genetic alterations have been identified in the mTOR pathway or chromatin modifying complexes such as PBRM1, SETD2, BAP1 among others^11^.

The genetic characterization of ccRCC tumors supported the development of an array of targeted therapies inhibiting the AKT-mTOR pathway^12^ or HIF target genes (particularly VEGF)^13^. Moreover, additional treatments such as HIF2α inhibitors^14^ or immune checkpoint inhibitors^15^ are under investigation. While such therapies led to significant advances in clinical responses and survival, important questions remain in terms of novel targets, predictive biomarkers, stratification of patients, and combination therapies.

By combining computational approaches and functional genomic datasets, we here identify PAX8 as a candidate oncogenic transcription factor in RCC regardless of VHL status. Characterization of its transcriptional function identifies a subset of metabolic genes regulated by PAX8 via enhancer elements. We focus on the ferroxidase ceruloplasmin (CP) as a prototype target gene in this category and dissect PAX8 molecular functions in controlling CP expression via a distal intragenic enhancer element. Importantly, CP is a predictive biomarker for PAX8 activity and its high expression identifies a subset of RCC patients with low survival. Hence, our data further support the concept of targeting PAX8 as a critical node for RCC proliferation and validates CP as a functional readout of PAX8 function.

## Results

### PAX8 is an oncogenic transcription factor in RCC

**Fig. 1:**
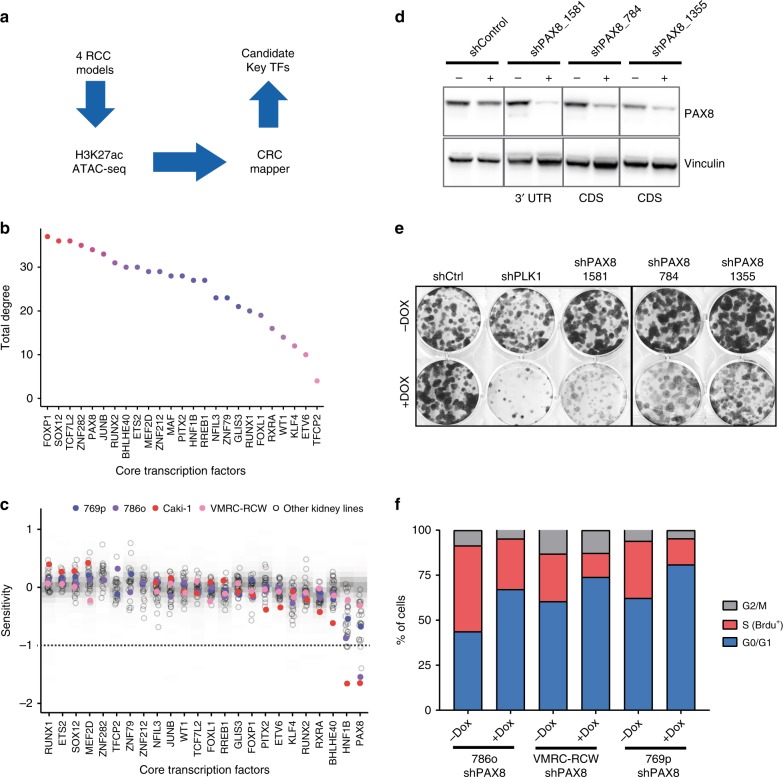
Core regulatory circuitry analysis identified PAX8 as a candidate oncogene in RCC. **a** Diagram of the experimental/computational pipeline to identify critical TFs in RCC. **b** CRC mapper output as TFs (*x*-axis) ranked by total degree (*y*-axis) in RCC. Total degree is a measure of a TFs’ contribution based on how often a given TF can participate in a regulatory interaction with the other TFs. **c** Sensitivity profile for CRC TFs (*x*-axis) in combined shRNA datasets (DRIVE, Achilles, Marcotte), where smaller values indicate that the cell line is more dependent on a given gene. Empty dots represent the sensitivity score for each TF in a RCC cell line. Colored dots represent the cell lines originally used in the CRC mapping. Shaded gray represents the distribution of sensitivity values for each TF in cell lines from other lineages. Horizontal dashed line at −1 represents the threshold for significant dependency. **d** Western blot validation of PAX8 knockdown efficacy in 786o cells infected with dox-inducible shRNAs. **e** Colony formation assay of 786o cells bearing various doxycycline-inducible shRNAs performed during 10 days. PLK1 is an essential gene in 786o cells and it is a positive control in this assay. **f** Cell cycle analysis by flow cytometry using PI-BrdU labeling in RCC cell lines bearing shPAX8_1581 upon 4 days of treatment with doxycycline

In order to characterize the landscape of active enhancers and promoters in kidney cancer cells we conducted ChIP-sequencing for H3K27ac and ATAC-sequencing of four ccRCC cell lines (Fig. 1a). We identified an average of ~23,800 H3K27ac sites and ~100,000 open chromatin regions across our four models (Supplementary Fig. 1A). In order to identify critical enhancer elements and core TFs that maintain the oncogenic state, we computed super-enhancers/core regulatory circuitries as previously described^16^.

Our analysis identified 25 candidate TFs that were prioritized by total degree, which is higher for the critical nodes within the top networks (Fig. 1b). In order to substantiate our findings, we evaluated the expression pattern as well as the cancer dependency of each candidate transcription factor as output of CRC mapper. As previously described, CRCs display higher expression compared with housekeeping genes^16^. Taking advantage of the expression profiling of the cancer cell line encyclopedia (CCLE) collection^17^, we ranked the candidates by expression levels (Supplementary Fig. 1B) and their scoring in genome-scale shRNA and CRISPR screens^18,19^ (Fig. 1c and Supplementary Fig. 1C). Interestingly, HNF1B and PAX8 were both highly expressed and sufficient to impact proliferation/survival of kidney cancer cell lines, making them prime candidates for downstream analyses. Due to the pattern of PAX8 expression in early embryonic kidney, its lineage potential for RCC oncogenesis^20,21^ and its dispensable role in normal kidney development (discussed below), we sought to focus on PAX8.

We then started to validate our findings in cellular models by generating stable cell lines with inducible shRNAs against PAX8. We cloned several shRNAs of which one (named sh1581) resulted in particularly efficient knockdown of PAX8 at the RNA and protein level (Fig. 1d). Importantly, the extent of PAX8 downregulation by the different shRNAs corresponded to the decrease of proliferation of the cells upon doxycycline treatment as measured by colony formation assay (Fig. 1e). PAX8 knockdown induced an accumulation of cells in the G1-S phase of the cell cycle and a concomitant reduction of BrdU labeled cells indicating decreased proliferation (Fig. 1f)^22^. To discard possible off-target effects induced by the shRNAs, we reintroduced in sh1581 cells a cDNA expressing full-length PAX8 (Supplementary Fig. 1D). Such cDNA escapes the shRNA-mediated downregulation since the sh1581 hairpin targets the 3′ UTR of PAX8. In the context of ectopic cDNA expression, downregulation of endogenous PAX8 failed to decrease proliferation in culture (Supplementary Fig. 1D). We further validated our findings with an orthogonal technology by generating Cas9-engineered cells bearing a dox-inducible sgRNAs against PAX8. Similarly to shRNAs, DOX induction led to strong PAX8 downregulation (Supplementary Fig. 1E) as well as decreased colony formation in 2D (Supplementary Fig. 1F).

In summary, CRC analysis combined with genome-scale genetic screens identify PAX8 as a candidate oncogenic factor in kidney cancer cells.

### PAX8 binds to regulatory elements linked to metabolic genes

**Fig. 2:**
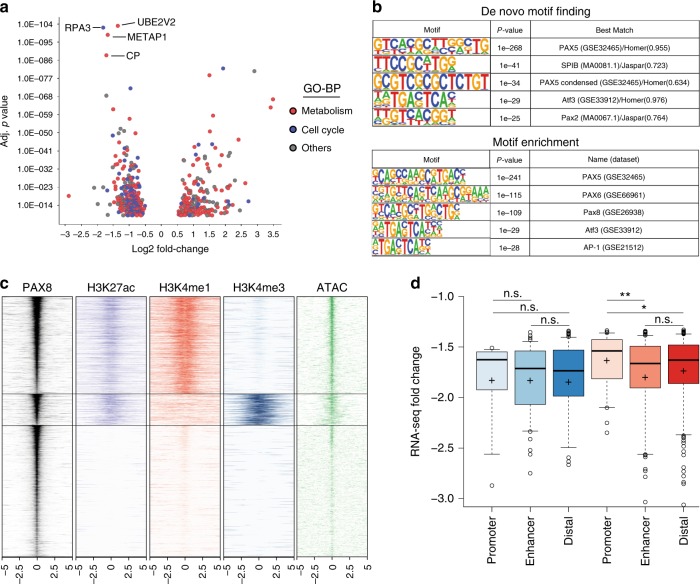
PAX8 regulates metabolic genes via enhancer elements. **a** Scatter plot representing the LogFC vs. Adj *p*-value of genes differentially expressed upon PAX8 knockdown across four RCC cell lines. Dots are color coded based on “Gene Ontology—Biological process” annotation of each gene. Blue represent genes involved in cell cycle and red represent genes consolidated from multiple metabolic process categories. **b** Slogos representation of Motifs enriched in PAX8 peaks by de-novo motif finding or motif enrichment. **c** Heatmap for PAX8, H3K27ac, H3K4me1, H3K4me3 ChIP-seq, and ATAC-seq signal around a 10 Kb window centered on PAX8 peaks. **d** LogFC as measured by RNA-seq upon PAX8 knockdown for genes positively regulated by PAX8 and categorized by bearing a PAX8 peak in the promoter, enhancer or distal site. Shades of blue represent genes in the category “Cell cycle”, while shades of red represent genes in the category “Metabolism” as per Fig. 2a. Center lines show the medians and crosses represent the mean; box limits indicate the 25th and 75th percentiles; whiskers extend 1.5 times the interquartile range from the 25th to 75th percentiles, outliers are represented by dots. *n* = 15, 91, 136, 53, 191, 359 sample points.*p < 0.05 and **p < 0.01 based on Welch’s T-test

In order to characterize the transcriptional program imposed by PAX8 in kidney cancer cells, we sought to quantify gene expression changes by RNA-seq upon PAX8 knockdown across multiple RCC models (Fig. 2a). Differential gene expression analysis across the four cell lines models identified 461 genes to be regulated in a PAX8-dependent manner (cutoff fold-change of 1.5 and adjusted *p*-value < 10^–10^) (Supplementary Fig. 2B). To control for off-target effects, we additionally performed RNA-seq in 786o cells with ectopic expression of PAX8 cDNA (as in Supplementary Fig. 1D) and observed dramatic attenuation of gene expression changes exerted by sh1581 (Supplementary Fig. 2C). Among differentially expressed genes, we observed several genes involved in kidney cell identity (i.e., Kidney Specific Cadherin CDH16 or Claudins CLDN1 and CLDN16) (Supplementary Fig. 2B), consistent with the notion of PAX8 controlling kidney cell fate and pathogenesis. A large fraction of deregulated genes are involved in cell cycle (i.e., RPA in Fig. 2a), consistent with decreased viability of RCC cells upon PAX8 knockdown and as previously reported^23,24^. Interestingly, we noticed a significant enrichment of genes involved in diverse metabolic processes, such as protein modifications and iron oxidation (e.g., UBE2V2 and CP), according to Gene Ontology categories (Fig. 2a).

In order to identify the subset of genes directly controlled by PAX8, we sought to characterize the genome wide occupancy of PAX8 across the same panel of kidney cancer cells. First, we identified a suitable antibody that could efficiently immunoprecipitate PAX8 upon crosslinking (Supplementary Fig. 2D). Since validated PAX8 target genes are exclusively expressed in thyroid cells (e.g., TG, TPO, and SLC5A5)^25^, we used the PAX8 promoter itself as a positive control for our ChIP assays, as previously reported^26^ (Supplementary Fig 2D, E). Our experiments identified 3634 PAX8 peaks shared by three out of four cell lines and used this dataset for further analysis. De novo TF motif finding in the sequences underlying PAX8 peaks readily identified a motif highly similar to the PAX motif (Fig. 2b). Indeed, HOMER motif enrichment analysis identified multiple PAX motifs (from the PAIRED domain) available in the Jaspar database as significantly enriched in PAX8-bound regions (Fig. 2b).

PAX8 was previously reported to be a transcriptional activator binding to the histone acetyltransferase p300 via its transactivation domain^25^. Accordingly, we examined PAX8 peaks for the presence of H3K27ac and categorized them as enhancers or promoters based on ChIP-seq data for H3K4me1 or H3K4me3. Hierarchical clustering of PAX8 peaks identified a large fraction of PAX8^+^/H3K27ac^+^-binding sites to belong mostly to enhancer elements characterized by the presence of H3K4me1 and devoid of H3K4me3 (Fig. 2c and Supplementary Fig. 2F–H). Importantly, when genes positively regulated by PAX8 were categorized according to the presence of a PAX8-binding site at their promoter or at enhancers, we observed stronger downregulation of the enhancer-controlled genes upon PAX8 knockdown compared with promoter-bound genes (Fig. 2d). This effect is specific to metabolic genes, corroborating the functional relevance of PAX8 regulation of such genes from enhancer elements.

### PAX8 regulates CP via a transcriptional enhancer element

**Fig. 3:**
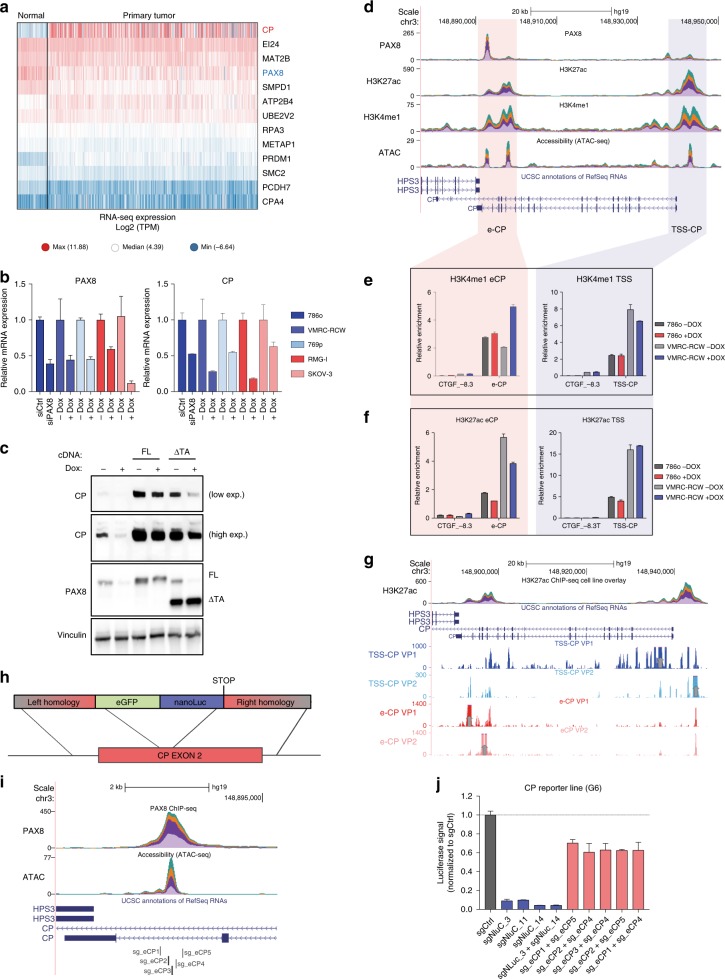
PAX8 regulates CP by favoring histone acetylation at its intragenic enhancer. **a** Heatmap of RNA-seq expression of the indicated genes (row) from TCGA dataset divided between normal (*n* = 104) and primary tumor (*n* = 823) samples. **b** qPCR for PAX8 (left) and CP (right) in different cell lines upon PAX8 silencing by doxycycline induced shRNA or siRNA. Shades of blue represent RCC cell lines while shades of red represent ovarian carcinoma models. Representative experiment of biological triplicates, error bars represent standard deviation of technical duplicates. **c** Western blot analysis of PAX8 and CP in 786o cells upon PAX8 knockdown and/or overexpression of a full-length (FL) PAX8 cDNA or PAX8 lacking the c-terminal transactivation domain (∆TA). Vinculin is used as a loading control. **d** UCSC genome browser tracks of overlaid stacked signal (one color for each of the four cell lines) for PAX8, H3K27ac, H3K4me1, and ATAC-seq centered at the CP locus. Red and Blue vertical shades indicate CP enhancer (e-CP) and CP TSS (TSS-CP) loci respectively. **e** H3K4me1 ChIP-qPCR at the e-CP (left) and TSS-CP (right) loci in 786o and VMRC-RCW cells upon doxycycline-inducible knockdown of PAX8. CTGF −8.3 locus is used as a negative control region. **f** H3K27ac analysis at the e-CP and TSS-CP loci, similar to Fig. 3e. Representative experiment of biological quadruplicates. Error bars represent standard deviation of technical triplicates. **g** UCSC genome browser tracks for 4C signal centered around the CP locus. The viewpoints for each track are indicated with vertical gray arrows. **h** Schematic diagram for the integration of a reporter cassette into the exon 2 of the CP gene in 769p cells. **i** UCSC genome browser snapshot representing the location of sgRNAs flanking the e-CP site used in the experiment shown in Fig. 3j. **j** Luciferase signal of 769p-CP reporter line (clone G6 from Supplementary Fig. 3D) transfected with the sgRNA combinations indicated on the *x*-axis. Error bars represent standard deviation of biological triplicates

In order to characterize how PAX8 might regulate the expression of metabolic genes, we focused on the top regulated genes to which a PAX8 peak was assigned. CP was of particular interest due to its dramatic overexpression in RCC cases compared with normal tissues as evidenced in the TCGA dataset (Fig. 3a). Among the target genes of PAX8, CP is the most dysregulated gene (Fig. 3a) and this effect is irrespective of relevant genetic aberrations such as VHL mutations (Supplementary Fig. 3A). In addition, tumors with different levels of CP expression display similar mutation frequency in most frequently mutated genes in RCC (Supplementary Fig. 3B).

We readily validated the regulation of CP upon PAX8 knockdown (with siRNA and shRNA) by qRT-PCR across multiple cell lines of renal and ovarian lineage (Fig. 3b). We also observed the downregulation of CP upon PAX8 knockdown at the protein level. Importantly, ectopic expression of full-length PAX8 reverted this effect, whereas a transactivation-domain-deficient PAX8 could only partially rescue CP expression (Fig. 3c and Supplementary Fig 3C). This evidence strongly suggests that a functional PAX8 is required for the activation of gene expression.

Interestingly, inspection of PAX8 ChIP-seq tracks revealed binding at two different sites near the CP transcriptional unit (Fig. 3d). While one PAX8 peak is around the TSS of the CP gene (named hereafter TSS-CP), the other PAX8-binding region identified is found near the 3′ end of the CP gene, at ~40 Kb from its TSS (named hereafter e-CP). As this latter site is also 3′ of the neighboring HPS3 gene (also around 40 Kb distance), the complex pattern of PAX8 binding prompted us to further characterize PAX8-dependent regulation of the CP-containing locus.

Interestingly, e-CP displayed features of an active enhancer element being positive for H3K27ac, H3K4me1 and transposase accessible, while being negative for the promoter mark H3K4me3 (Fig. 3d). Due to the reported interaction between PAX8 and p300, we characterized the impact of PAX8 knockdown on histone acetylation at the e-CP locus. Importantly, while H3K4me1 was unaffected upon PAX8 knockdown (Fig. 3e), we observed a reduction in H3K27ac at the PAX8 e-CP site corroborating the notion that PAX8 can recruit an acetyltransferase-containing complex for gene activation (Fig. 3f).

In order to dissect the role of the PAX8-bound enhancer element of CP, we first analyzed the proximity of such enhancer to related promoters by circular chromosome conformation capture (4C-seq). Using either the e-CP or the TSS-CP as viewpoints, we observed reciprocal contacts between the two PAX8 containing sites suggestive of the role in the e-CP locus to control the activity of the CP promoter (Fig. 3g).

To functionally validate the role of the e-CP site, we generated a CP reporter cell line by introducing a NanoLuc-eGFP fusion cassette into intron 2 of CP using CRISPR-Cas9 (Fig. 3h).

Upon cotransfection of a sgRNA targeting the exon 2 of CP and the repair template in cells expressing Cas9 and doxycycline-inducible sh1581, we could FACS-sort a number of clones expressing NanoLuc under the CP promoter. Moreover, knockdown of PAX8 by inducible shRNAs induced a decrease in NanoLuc signal comparable to that of the endogenous CP gene (Fig. 3b and Supplementary Fig 3D).

We then transfected this reporter cell line with sgRNA pairs flanking the PAX8-binding site of the e-CP locus (Fig. 3i). While sgRNAs targeting NanoLuc drastically decreased the luciferase signal, sgRNAs targeting e-CP mildly decrease luciferase activity, paralleling the downregulation observed with shPAX8 (Figs. 3j and b). Importantly, sgRNAs against e-CP did not affect the luciferase signal in a cell line bearing the NanoLuc-eGFP cassette driven by a constitutive promoter (Supplementary Fig. 3E).

Collectively our data demonstrate that PAX8 can activate gene expression by controlling the activity of distal enhancer elements such as e-CP.

### PAX8 regulates CP and Fe^3+^ levels across RCC models

**Fig. 4:**
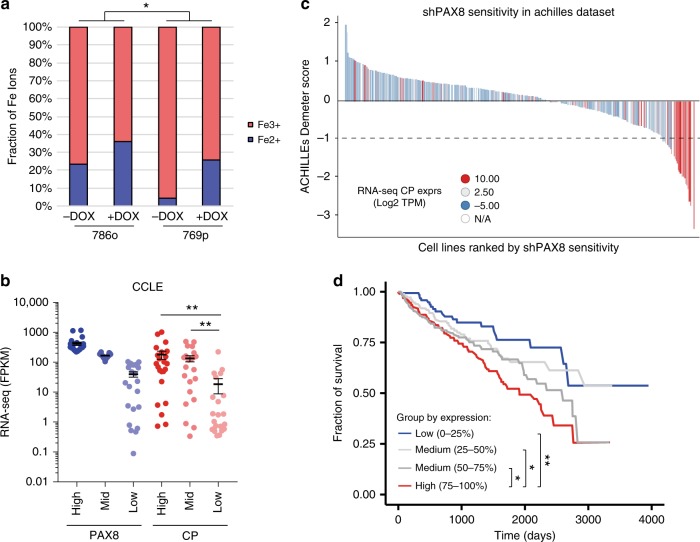
CP expression tracks with PAX8 sensitivity and RCC cases with poorer survival. **a** Iron assay kit measurements of different iron oxydation states in 786o and 769p cells conditioned media upon PAX8 knockdown. *p < 0.05 based on ANOVA test. **b** RNA-seq expression of PAX8 and CP across the cancer cell line encyclopedia segregated evenly according to PAX8 High, Mid, and Low expression. **p < 0.005 based on Welch’s T-test. **c** Waterfall plot representing the sensitivity (Demeter score) of cell lines to PAX8 knockdown as reported in the Achille’s project dataset. Each histogram bar is a cell line and bars are color coded based on the CP expression level (by RNA-seq). **d** Kaplan–Meyer plot representing the survival of TCGA RCC cases segregated based on CP expression (4 equal-sized bins). *p < 0.05 and **p < 0.01 according to Mantel–Cox log-rank test

After establishing CP as a downstream target gene of PAX8, we wanted to understand the functional relevance of CP transcript regulation. We then analyzed conditioned media from cell lines bearing doxycycline-inducible shPAX8 and observed that knockdown of PAX8 led to a reduction in the level of CP secreted in the media (Supplementary Fig. 4A). This, in turn, translated into a perturbation of relative levels of oxidized iron. In particular, we observed a decrease in Fe^3+^ (catalyzed by CP) in favor of an accumulation of the ferrous ion Fe^2+^ (Fig. 4a).

PAX8-dependent CP regulation appears to be applicable to a large cohort of models. In fact, segregation of the CCLE RCC and ovarian models based on PAX8 expression revealed low expression of CP in cellular models expressing low PAX8 (Fig. 4b). Such concept held true also in vivo, as shown by RNA-seq analysis of our internal RCC patient-derived xenografts collection, which displayed remarkably low expression of CP in models bearing low PAX8 expression (Supplementary Fig. 4B).

CP expression not only correlates with PAX8 expression but also with PAX8 dependency. Indeed, cell lines bearing high CP expression displayed sensitivity to PAX8 knockdown in two large-scale shRNA datasets suggesting that CP might be a surrogate marker for PAX8 activity (Fig. 4c and Supplementary Fig. 4C). Importantly, when probing the biomarker potential for CP in the TCGA dataset, we observed that RCC cases bearing high expression of CP display lower survival compared with the low expressers (Fig. 4d) while PAX8 mRNA expression does not seem to have prognostic value (Supplementary Fig. 4D). These data propose CP as a marker for aggressive/refractory RCC and suggest PAX8 activity as relevant for RCC progression and aggressiveness supporting the notion of PAX8 as a potential therapeutic target for RCC.

## Discussion

In this study, we identify PAX8 as a cell autonomous oncogenic driver in RCC through CRC analysis and functional genomics. In addition, we characterize the epigenomic landscape controlled by PAX8 in RCC cells and show that PAX8 activates a large set of genes involved in cell cycle and metabolism mainly through distal enhancer elements. Moreover, we show that PAX8 is required for establishing H3K27Ac at a subset of its genomic binding sites, which is associated with activation of target genes like CP. Importantly, we show that the expression of CP can distinguish a subset of RCC patients that have poor outcome independently of known genetic aberrations.

Mapping core regulatory circuitries has become a valuable method to identify critical TFs for cell identity as well as potential therapeutic nodes in cancer^7,16^. While the CRC mapper output consisted of multiple TFs, intersection with transcriptomic and functional genomic datasets highlighted the importance of PAX8 and HNF1B in cancer cells proliferation. However, since the genetic screens analyzed rely on in vitro cellular representation as readout^18,19^, we cannot exclude that other TFs identified by CRC mapper might be essential for other RCC processes in vivo such as cellular differentiation etc.

HNF1B is a well-known master regulator of tissue specification. HNF1B is expressed during early embryonic development and adulthood in a variety of tissues such as kidney, pancreas, and liver^27^. In early kidney development, HNF1B controls nephron patterning^28^, ureteric bud branching, and collecting duct morphogenesis^29^, supporting its role as master regulator of renal cells identity. However, HNF1B mutations/deletions have been shown to lead to a variety of renal malignancies such as polycystic kidney disease^30,31^ or even supporting chromophobe renal cell carcinoma development^32^. Such data prompted us to prioritize instead PAX8 as a potential target in ccRCC.

Previously, PAX8 was identified as a critical regulator of thyroid and kidney development. *Pax8* knockout mice die postnatally due to defects in the maturation of follicular thyroid cells. Interestingly, this phenotype can be reverted by administration of the thyroxine hormone^33^. In addition, germline mutations in PAX8 DNA-binding domain in humans (abolishing PAX8 transcriptional activity), lead to hypoplasia and a compensatory thyroid hyperactivity^34^.

However, the expression pattern of PAX8 during development reveals a broad, yet selective, expression pattern in renal proximal tubule cells (PTC) and fallopian tube secretory epithelial cells (FTSEC). Indeed, *Pax8* knockout male embryos display an overall normal urogenital tract^35^.

Interestingly, strong genetic interaction in morphogenic development of the proximal tubule is observed upon hemizygous inactivation of *Pax2* (which is a paralog of *Pax8*)^35^. This data indicates that PAX8 is largely dispensable for adult tissue homeostasis, suggesting a high therapeutic index for modulators of PAX8 functions in the context of cancer. Moreover, the widespread expression of PAX8 in the majority of renal, ovarian and endometrial cancer cases (it is used as a diagnostic marker for such diseases^21^), suggest a large population potentially benefitting from PAX8-based therapies.

The restricted expression pattern of PAX8 was exploited for lineage tracing experiments to determine the cell of origin of cancer. Indeed transgenic mice with reporter alleles display specific expression of PAX8 in adult PTC and FTSEC^20,36^. Importantly, VHL inactivation^36^, when combined with other genetic lesions in Pax8-expressing cells, leads to neoplastic lesions of different stage and penetrance^37,38^ suggesting proximal tubules as a potential source of RCC.

Similarly, tumors resembling high-grade serous ovarian cancer (HGSOC) are observed when prototypical ovarian cancer lesions are induced in PAX8-expressing FTSEC^39^. These mouse models demonstrate that PAX8-expressing cells might be the cell of origins of RCC and HGSOC. To date there are no models available for conditional inactivation or ectopic expression of PAX8, which hampers the understanding of sufficiency and dependency of PAX8 for tumorigenesis.

PAX8 knockdown leads to profound decrease of proliferation in RCC cells, most likely by regulating cell cycle genes as we and others reported^23,24^. However, our work revealed that PAX8 also regulates a large number of genes involved in metabolism. Along these lines, for many years, PAX8 has been suggested to be the master regulator of thyroid enzymes such as TPO^25^. Moreover, PAX8-dependent regulation of erythrocyte function has been previously reported by a gene ontology annotation of PAX8 targets in ovarian cancers^23^. This evidence suggests a potential therapeutic combination with the HIF pathway, as evidenced by the regulation of the prominent target gene Erythropoietin (EPO). Interestingly CP has been reported to be a HIF target gene^40^ and we additionally identified the EPO receptor RHEX (C1ORF186)^41^ as downregulated upon shPAX8 further supporting an iron-metabolic convergent pathway controlled by HIF and PAX8 in renal carcinogenesis.

The intertwining of transcriptional pathways is particularly appealing when considering the spatial organization of EPO/iron metabolism in kidneys. Indeed EPO production is generally stimulated by HIF factors in fibroblast-like interstitial cells^42^, while suppressed in tubular epithelium^43^. In the latter setting, instead, PAX8 drives expression of proteins such as CP and RHEX directly involved in iron homeostasis, further corroborating the importance of the tubulointerstitial cellular crosstalk.

To the best of our knowledge, our report is the first attempt to validate the oncogenic role of PAX8 in RCC and to characterize the role of PAX8 at enhancer elements. However, additional work is necessary to understand the molecular functions of PAX8 at repressed regions and its contribution to gene silencing. Future studies should also aim at characterizing the protein complexes engaged by PAX8 in different contexts (RCC vs. Ovarian and normal vs. cancer) in order to provide a rational for novel drugs that can inhibit the function of PAX8 in cancer cells.

## Methods

### Cell culture experiments

769p, 786o, and VMRC-RCW were maintained in RPMI (Gibco) supplemented with 10% FBS, 1X Hepes, 1X l-Glutamine. CAKI-1 cells were maintained in McCoy’s 5A with 15% FBS and 1X l-Glutamine. All cell lines were obtained from ATCC and tested for identity by SNP genotyping and mycoplasma contamination. Doxycycline-inducible shRNA cells were obtained by lentiviral transduction of pLKO-TET-ON constructs containing the following shRNA sequences: sh784 5′-ccgactaagcattgactcaca-3′; sh1355 5′-ggaagtgaatactctggcaat-3′; sh1581 5′-gagagtcacacaaaggaatct-3′ and shPLK1 5′-ggtatcagctctgtgataaca-3′. PAX8 constitutive overexpression was achieved by infecting cells with a pLNCX2 retroviral construct containing PAX8 Full length or PAX8 ∆TA (aa 1-328) cDNA between BglII and SalI sites. 786o_Cas9 and 769p_Cas9 were obtained by retroviral transduction with a lentiviral construct overexpressing spCas9 under EF1A promoter (named pNGX_LV_c028). Doxycycline-inducible sgRNA cells were obtained by lentiviral transduction of 786o_Cas9 cells with a modified pLKO-TET-ON construct containing the following sgRNA sequences: sgCtrl 5′-gatgagctcaatcggcactg-3′; sgPSMA3 5′-gatgagctcaatcggcactg-3′; sgPAX8 UTR/ATG 5′-ggcgatgcctcacaactcacaactcc-3′ and sgPAX8 Exon3 5′-tccatggcctaaggagaca-3′. Transfections were performed with Lipofectamine RNAi MAX (Invitrogen) according to manufacturer’s recommendation. siCtrl is All star Negative Control siRNA (QIAGEN) and siPAX8 is a human Dharmacon PAX8 Smart Pool.

CP reporter line was generated by cotransfection of 769p_Cas9_sh1581 cells with the following sgRNAs (annealed synthetic crRNA with TracrRNA (IDT)) against exon 2 of CP (CP_guide_1 5′-caactatagaaaaaccggtc-3′, CP_guide_3 5′-atgaaaggtgtagggcctag-3′) and a repair template encompassing 800 bp upstream and downstream of the cleavage site flanking an in-frame cassette encoding eGFP fused to NanoLuc sequence. Positive signal cells were retrieved by FACS on a Sony Flow Cytometer model SH800S.

CP-reporter modulation was evaluated 72 h after Doxycycline treatment or after transfection with combinations of the following sgRNAs: sg_eCP1 5′-TCTTTGGCCCATAAAATCAG-3′; sg_eCP2 5′-GATTACTGCTTGGTTAAGAA-3′; sg_eCP3 5′-CATGCAGAGTATGGCACTAC-3′; sg_eCP45′-TTGCATGTAAACCACACAAA-3′; sg_eCP5 5′-CAGTGACTGCTCTAATAAAA-3′; sgNluC_3 5′-GGTCTGAGCGGCGACCAAAT-3′; sgNluC_11 5′-GAGCGCCTGATCAACCCCGA-3′ and sgNluC_14 5′-GATGGTTACTCGGAACAGCA-3′.

Luciferase signal was measured using NanoGlo Luciferase Assay system (Promega), Iron oxidation was measured in conditioned media using Iron Assay Kit (Abcam Ab83366) according to manufactured recommendation.

Cell cycle profile was measured by flow cytometry (Beckman Coulter CytoFLEX S) with cells pulse-labeled with BrdU and stained with Propidium Iodide and BrdU-FITC antibody (Cell Signaling, 5292; 1:200 dilution).

All the other gene expression and chromatin-related experiments in shPAX8 cells were performed after 4 days treatment with 100 ng/ml of doxycycline unless indicated otherwise. Colony formation assays were performing seeding cells at low density (2500 cells/well in 6-well plates) with continuous treatment with doxycycline for 10 days, after which cells were fixed with 4% formaldehyde for 10 min and colonies stained with crystal violet.

### Gene expression analyses

For immunoblotting, cells were harvested and lysed in RIPA buffer supplemented with protease inhibitor cocktail (Roche). Protein samples were resolved on SDS-PAGE, transferred onto nitrocellulose membranes and probed with the following antibodies: PAX8 (Cell Signaling, 59019; 1:1000 dilution), CP (Cell Signaling, 98971; 1:1000 dilution), Vinculin (Sigma, V9131; 1:400 dilution). Uncropped original western blot images are provided in the supplementary material.

For quantitative PCR, total RNA was extracted from cell pellets using RNAeasy Mini kit Plus (QIAGEN) according to the manufacturer’s instructions. qRT-PCR was performed with QuantStudio 6 Flex (Applied Biosystems) using master mix reagent (Roche). Gene expression levels were normalized to the HPRT housekeeping gene. For ChIP-qPCR, qPCRs were performed using the Fast SYBR green master mix reagent (Applied Biosystems).

### 4C sequencing

4C templates were prepared according to a previously published protocol^44^. Briefly, DpnII digestion was used as the first restriction enzyme to generate high-resolution 3C template, which was further trimmed with Csp6I, NlaIII or BfaI. 4C primers were design following the general consideration as described^44,45^. The primers (TSS-CP VP1_Fw: 5′-GTTTAAGCTGAATGTTGATC-3′; TSS-CP VP1_Rv: 5′-GCCGCACACATCTATGCAAAAAG-3′; TSS-CP VP2_Fw: 5′-TCCCTTTTGCATACGAGATC-3′; TSS-CP VP2_Rv: 5′-CGACCCACCTGATAAGTCAAAGT-3′; e-CP VP1_Fw: 5′-TTTTGTTTCTTCCCTGGATC-3′; e-CP VP1_Rv: 5′-CCGACCTTTAGTGCAAATGGAAA-3′; e-CP VP2_Fw: 5′-TTCCACCAGAATGAAGGATC-3′; e-CP VP2_Rv: 5′-GAATCTTGTTTCCAGGACCTCTA-3′) carried additional 5′ overhangs composed of adaptor sequences for Illumina single-read sequencing. Samples were sequenced on a Mi-Seq machine (Illumina).

### ChIP-seq, RNA-seq, and ATAC-seq

ChIP-seq was performed as previously described^46^. Cells were cross-linked in 1% formaldehyde for 10 min at room temperature after which the reaction was stopped by addition of 0.125M glycine. Cells were lysed and harvested in ChIP buffer (100 mM Tris at pH 8.6, 0.3% SDS, 1.7% Triton X-100, and 5 mM EDTA) and the chromatin disrupted by sonication using a EpiShear sonicator (Active Motif) to obtain fragments of average 200–500 bp in size. Suitable amounts of chromatin were incubated with specific antibodies overnight. Antibodies used are: PAX8 (Cell Signaling, 59019), H3K27ac (Cell Signaling, 8173), H3K4me1 (Cell Signaling, 5326), and H3K4me3 (Millipore 07–473). Immunoprecipitated complexes were recovered on Protein G Dynabeads (Invitrogen) and, DNA was recovered by reverse crosslinking and purified using SPRI Select beads (Beckman Coulter). Libraries for ChIP-sequencing were generated using Ovation^®^ Ultralow Library System V2 (NuGEN) and barcodes were added using NEBNext Multiplex Oligos for Illumina (Index Primers Set 1) (NEB) according to the manufacturer’s recommendation. RNA-seq libraries were generated using TruSeq RNA Sample Prep Kit v2 (Illumina) according to manufacturer’s recommendation. ATAC-seq was performed as previously described^47^.

All next-generation-sequencing experiments were run on a HiSeq2500 (Illumina) sequencer.

### ChIP-seq data processing

ChIP-seq data were mapped to the human reference genome (hg19 assembly) using bwa 0.7.8^48^ with following parameters bwa aln -n 2 and bwa sampe -n 5. Mapped reads with a mapping quality lower than 25 were disregarded. Duplicated reads were removed using the MarkDuplicates utility of the Picard tools (http://broadinstitute.github.io/picard) and peaks called using macs2 version 2.1.0^49^ with the following parameters --bw 500 -m 5 50 -p 0.00000001.

For global analyses of PAX8-binding sites, a PAX8 ChIP-seq consensus peakset was defined as peaks identified in three out of four cell lines. We then fixed this peakset as reference for the three histone marks (H3K27ac, H3K4me1, and H3K4me3) as well as ATAC-seq. The consensus PAX8 peaks were centered to the cell line specific peak summits. Normalized read counts for 10,000 bases in bins of 10 bases from the summit centers were collected for clustering. The hierarchical clustering was performed on the three epigenomics mark count data using R functions dist (Euclidean distance) and clust (Ward’s D2 method). The clustered data were cut in three clusters to capture three potential chromatin states (combinations of H3K27ac±, H3K4me1±, and H3K4me3±). The full ChIP/ATAC-seq dataset was then plotted over a 4 kb window centered at summits and organized according to the three clusters. The clusters and heat maps were generated with the genomation R/Bioconductor package interface^50^. Motif finding was performed using Homer (http://homer.ucsd.edu/homer/motif/) with default parameters. Raw data have been deposited to Array Express under accession E-MTAB-7812

### RNA-seq data processing

Gene-level expression quantities were estimated by the Salmon algorithm^51^. Differential expression analysis was performed with DESeq2^52^ and genes with LogFC cutoff >0.6 and Adj. *p*-value > 10^–10^ were selected for downstream analyses (Supplementary Data 1). Functional annotation of differentially expressed genes was performed using DAVID (https://david.ncifcrf.gov/) using the Gene Ontology Biological Process level 2 annotation. Genes belonging to either cell cycle or metabolic categories were then color-coded accordingly in the volcano plot. Genes were categorized based on the absolute distance from the closest PAX8 ChIP-seq peak as Promoter (<10 Kb), Enhancer (10 Kb < x < 400 Kb) or Distal ( >400 Kb). Raw data have been deposited to Array Express under accession E-MTAB-7808

### ATAC-seq data processing

ATAC-seq data were mapped and deduplicated as described for the ChIP-seq data. We used the Riesling pipeline (https://github.com/GordonLab/riesling-pipeline) to pre-process the data in two steps. We first used it to remove reads that were: PCR duplicates; mapped to the mitochondria DNA; mapped to a hg19 blacklisted region (https://sites.google.com/site/anshulkundaje/projects/blacklists); or had map quality <10. Next, we used Riesling to call peaks using Macs v.1.4^49^ with a peak *p*-value cutoff of 1 × 10^–9^. We stitched together the ATAC-seq peaks from the four cell lines and calculated their normalized read density using the Bamliquidator (version 0.2) read density calculator (https://github.com/BradnerLab/pipeline/wiki/bamliquidator). Raw data have been deposited to Array Express under accession E-MTAB-7844.

### Identifying active genes, super-enhancers, and open chromatin

For histone modification ChIP-seq, we stitched together the ChIP-seq peaks from the four cell lines and calculated their normalized read density using the Bamliquidator (version 0.2) read density calculator (https://github.com/BradnerLab/pipeline/wiki/bamliquidator). Briefly, reads aligning to stitched regions further than 2500 bp from a transcription start site were extended to 200 bp and the density of reads per base pair (bp) was calculated. The density of reads in each region was normalized to the total number of million mapped reads producing read density in units of reads per million mapped reads per bp (rpm/bp).

We identified super-enhancers using the rank ordering of super-enhancers (ROSE^53^) meta algorithm (https://github.com/BradnerLab/pipeline), excluding H3K27ac peaks within 2500 bp of a transcription start site and stitching together peaks from any RCC cell line within 1638 bp. We identified regions of open chromatin by merging overlapping ATAC-seq peaks for the four cell lines.

### Defining core transcription regulatory circuitry (CRC)

We performed the core transcriptional regulatory circuitry analysis as previously described^7,54^ using CRC (https://github.com/linlabcode/CRC) which required three files: a list of active genes; a list of super-enhancers; and nucleosome free regions. We identified active genes as Hg19 RefSeq genes with at least one H3K27Ac peak from one of the four RCC cell lines mapping to within 1000 bp of the transcription start site (14,239 active transcripts for 12,297 active genes). We identified super enhancers using the rank ROSE^53^ meta algorithm (https://github.com/BradnerLab/pipeline), excluding H3K27ac peaks within 2500 bp of a transcription start site and stitching together peaks from any RCC cell line within 1638 bp (926 super enhancers for 793 genes). Nucleosome free regions were defined as ATAC-seq peaks contained within the super-enhancers of active genes. Within these regions, the CRC software uses FIMO (Grant et al. 2011) to find enriched (*q* value < 1e−5) TF motif occurrences. CRC defines TF motifs using a custom database aggregating published TF motif position weight matrices from TRANSFAC^55^, JASPAR^56^ and from high throughput SELEX experiments^57^. CRC first identified 25 candidate TFs that are active, regulated by a proximal, overlapping, or the closest super enhancer region for which we have motif data in our custom database. For these candidate TFs, we defined a regulatory interaction as a TF binding to a nucleosome free region inside an active super-enhancer region. The total degree is a measure of how often a given TF participates in a regulatory interaction with other TFs. It is defined as the number of unique TFs participating in a regulatory interaction that affects a given TF plus the number of unique TFs that are regulated by a given TF.

### Functional genomics screening datasets

We downloaded the RPKM-normalized mRNA expression data (version 18q3) from the CCLE^17^ at https://portals.broadinstitute.org/ccle/data on 24 September 2018. The sensitivity profile for each gene’s response to RNAi was downloaded from https://figshare.com/articles/DEMETER2_data/6025238/2 (version DEMETER2 v2) on September 24, 2018, which was precomputed by applying the DEMETER2 algorithm^18^ to the combined data from Project DRIVE^19^, Project Achilles^18^, and 76 additional breast cancer cell lines^58^. The sensitivity score returned by the DEMETER2 algorithm was designed to distinguish between the on- and off-target effects of RNAi. The sensitivity score gives a measure of the statistical significance of the change of those shRNAs compared with the background of the rest of the shRNAs.

The CRISPR knockout dependency score from the Cancer Dependency Map was downloaded (https://figshare.com/articles/DepMap_Achilles_18Q3_public/6931364/1) on September 24, 2018 (version 18q3), which was generated as previously described^59^. Briefly, the CRISPR dependency score is an estimate of the probability that a cell line is dependent on a gene using the CERES algorithm, which controls for genomic copy number changes^59^.

### TCGA data representation

TCGA gene expression and mutations data were downloaded from https://portal.gdc.cancer.gov/ and the heatmap for RNA-seq expression of PAX8 target genes in RCC was generated using Tibco Spotfire https://www.tibco.com/products/tibco-spotfire.

For Kaplan–Meier curves, RCC cases were divided in four equal-sized bins based on either CP or PAX8 expression and survival curves were generated in R using the survival package https://cran.r-project.org/web/packages/survival/index.html. For mutation data, RCC cases expressing CP were divided in three equal-sized bins based on levels of CP expression and mutation status for ten most mutated genes in RCC was plotted.

### Reporting summary

Further information on research design is available in the Nature Research Reporting Summary linked to this article.

## Supplementary information

Supplementary Information

Description of Additional Supplementary Files

Supplementary Data 1

Reporting Summary

## Data Availability

The authors declare that the main data supporting the findings of this study are available within the article and its Supplementary Information files. Sequencing data are deposited in Array Express under accession numbers E-MTAB-7808, E-MTAB-7812 and E-MTAB-7844.
