# Supplementary material for: PAX8 activates metabolic genes via enhancer elements in Renal Cell Carcinoma
Source: Nat Commun. 2019 Aug 20;10:3739. doi: 10.1038/s41467-019-11672-1 (PMC6702156; doi:10.1038/s41467-019-11672-1)

SUPPLEMENTARY INFORMATION

Bleu M., Gaulis S., Lopes R. et al., PAX8 activates metabolic genes via enhancer elements in Renal Cell Carcinoma

A

	H3K27AC	ATAC-seq
769p	20,444	64,485
786o	20,075	94,682
Caki-1	26,658	126,552
WMRC-RCW	28,384	120,675

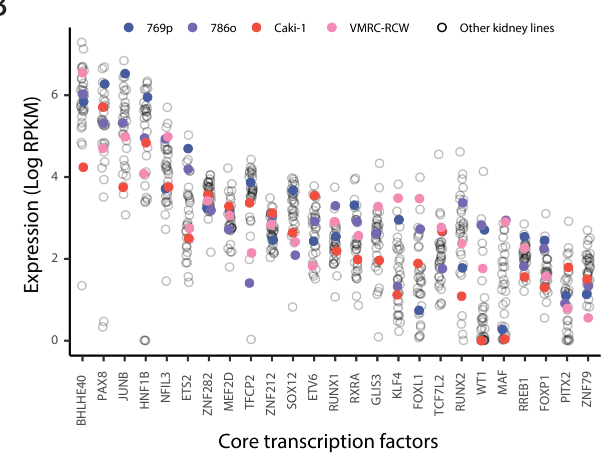

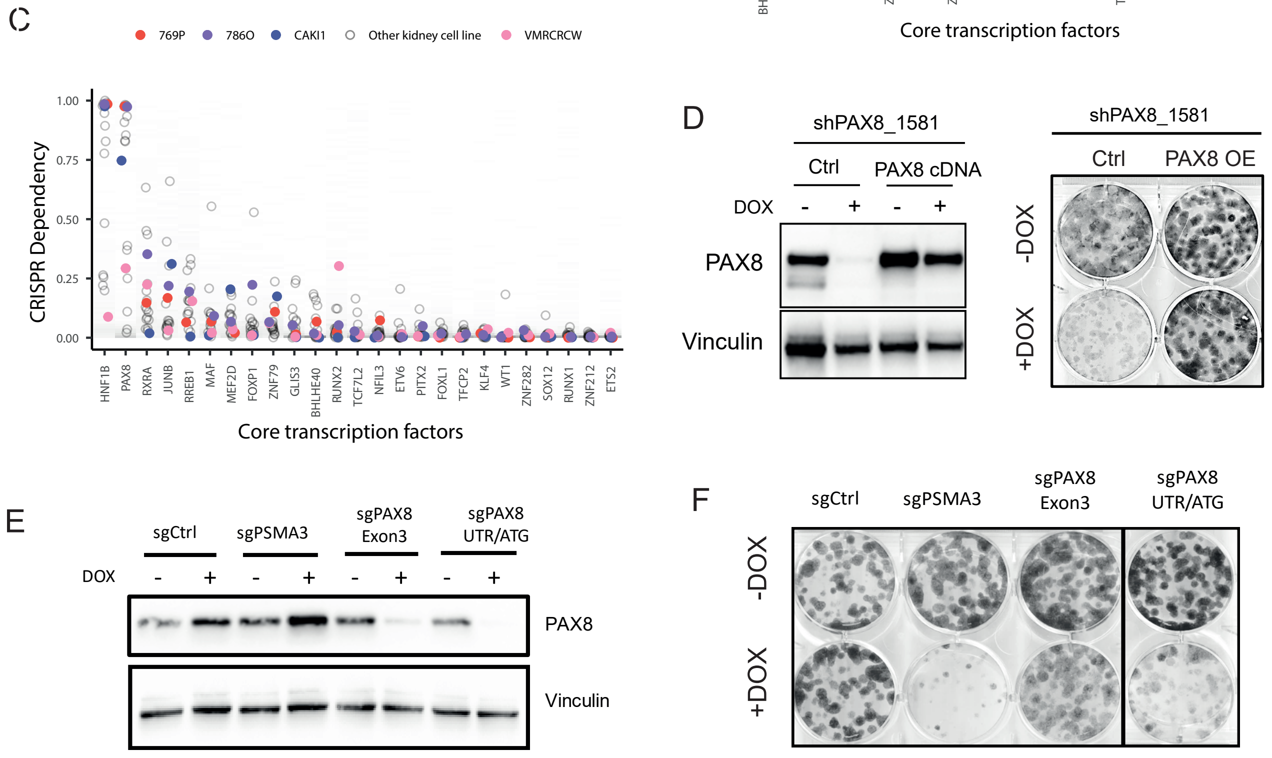

Supplementary Figure 1. Core Regulatory Circuitry supporting data and tools for genetic manipulation of PAX8

A) Table indicating the number of H3K27ac and ATAC-seq peaks across the four RCC models employed. B) RNAseq expression analyses for the indicated TFs (x-axis) across the CCLE dataset. Colored dots indicate the cell line models originally included in CRC analysis; empty dots indicate other RCC lines. C) CRISPR dependency score for CRC TFs in the Cancer Dependency Map project dataset. Each dot represents an estimate of the probability that a cell line is dependent on a gene measured by the CERES algorithm. Colored dots represent the cell lines originally used in the CRC mapping. D) Western blot (left) and Colony formation assay (right) for 786o cells infected with doxycycline-inducible shPAX8_1581 and/or constitutive ectopic expression of PAX8 full-length cDNA. E) Western blot analysis of 786o_Cas9 cells bearing doxycycline-inducible sgRNAs against PSMA3 (essential gene) or PAX8 treated with doxycycline or vehicle for 4 days. F) Colony formation assay of the cells from figure S1E treated with doxycycline or vehicle for 10 days.

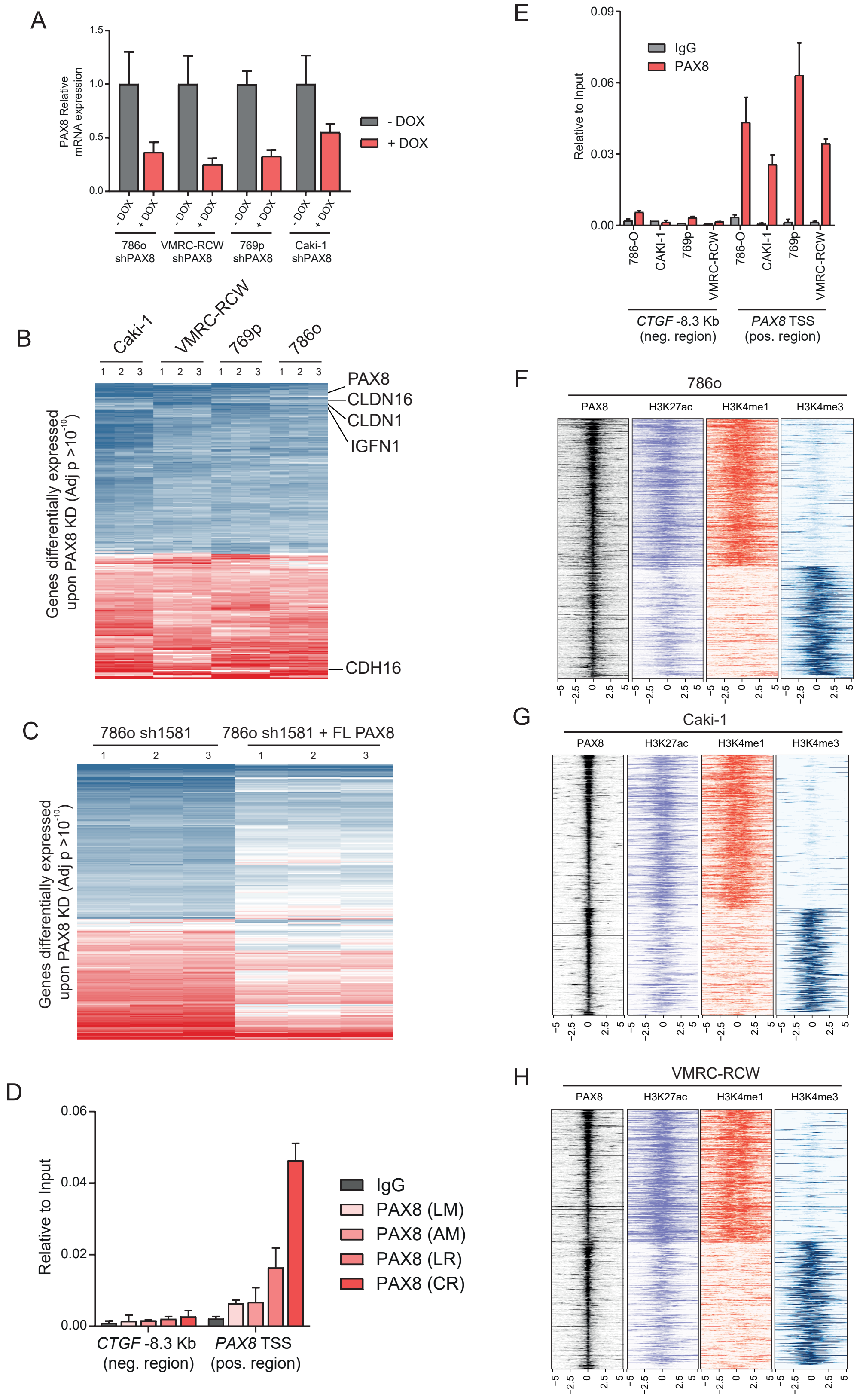

Supplementary Figure 2. RNA-seq and ChIP-seq data to characterize PAX8 target genes

A) qPCR validation for the effectiveness of PAX8 downregulation upon Doxycycline treatment (4 days) of four cellular models bearing doxycycline-inducible shPAX8. B) Heatmap of LogFC between control and doxycycline treament in four cell lines represented in figure S2A for differentially expressed genes as determined by RNA-seq analyses. Examples of genes involved in kidney cell identity are indicated. C) Heatmap of the LogFC for the same genes reported in figure S2B in an indipendent RNA-seq experiment in 786o_shPAX8 cells or 786o_shPAX8 cells with constitutive expression of full length PAX8 cDNA. D) ChIP-qPCR validation of four different PAX8 antibodies in 786o cells on a positive control region (PAX8 TSS) vs. a negative control region (CTGF -8.3). E) ChIP-qPCR validation of PAX8 (CR) antibody in four different RCC models. F-G-H) ChIP-seq heatmaps for PAX8, H3K27ac, H3K4me1 and H3K4me3 in PAX8+/H3K27ac+ sites in 786o (F), CAKI-1 (G) and VMRC-RCW (H) cells. Error bars represent standard deviations.

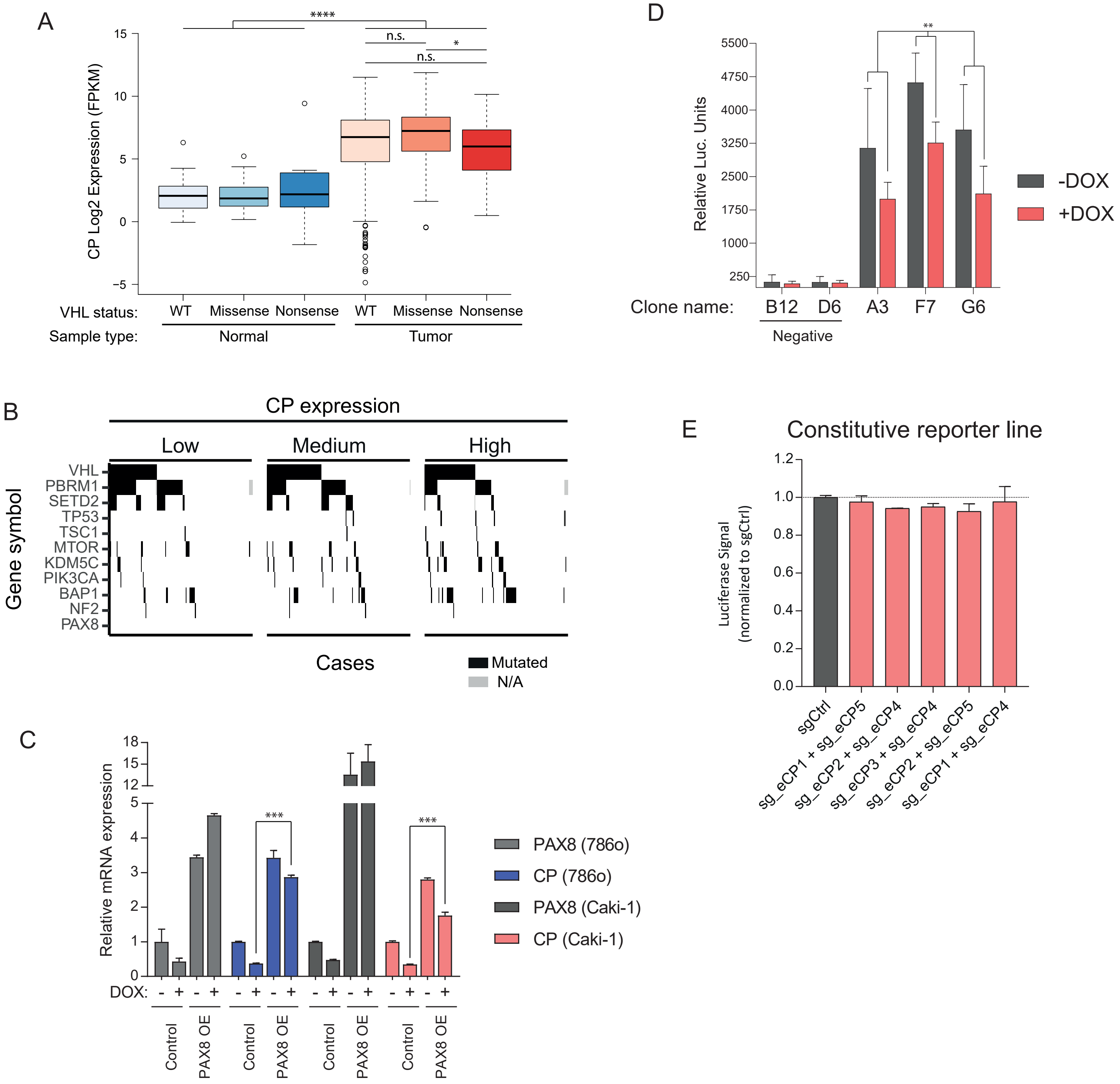

Supplementary Figure 3. PAX8-dependent CP regulation

A) RNA-seq expression for CP in TCGA-RCC dataset categorizing samples based on "Type" (Tumor vs. Normal) and type of mutations in VHL gene. Center lines show the medians; box limits indicate the 25th and 75th percentiles; whiskers extend 1.5 times the interquartile range from the 25th and 75th percentiles, outliers are represented by dots. n = 40, 21, 10, 325, 136, 41 sample points. * = p<0.05, **** = p<0.0001, n.s. = not significant based on Anova test B) Mutation status for the 10 most frequently mutated genes in RCC (y-axis) in RCC cases segregated in equal-size bins by CP expression level. C) qPCR for PAX8 and CP expression in two different cell lines infected with doxycycline-inducible shPAX8_1581 and/or constitutive ectopic expression of PAX8 full-length cDNA. PAX8 overexpression sustains significant levels of CP expression *** = p<0.001 by student T-test. D) Luciferase signal from different clones obtained from transfection of 769p_Cas9 transfected with sgRNAs against CP exon 2 and HR repair template to generate CP reporter line. ** = p<0.005, based on Anova test. E) Luciferase signal of a 769p_Cas9_EF1A_Luc cell line transfected with the indicated sgRNAs against CP. Error bars represent standard deviations.

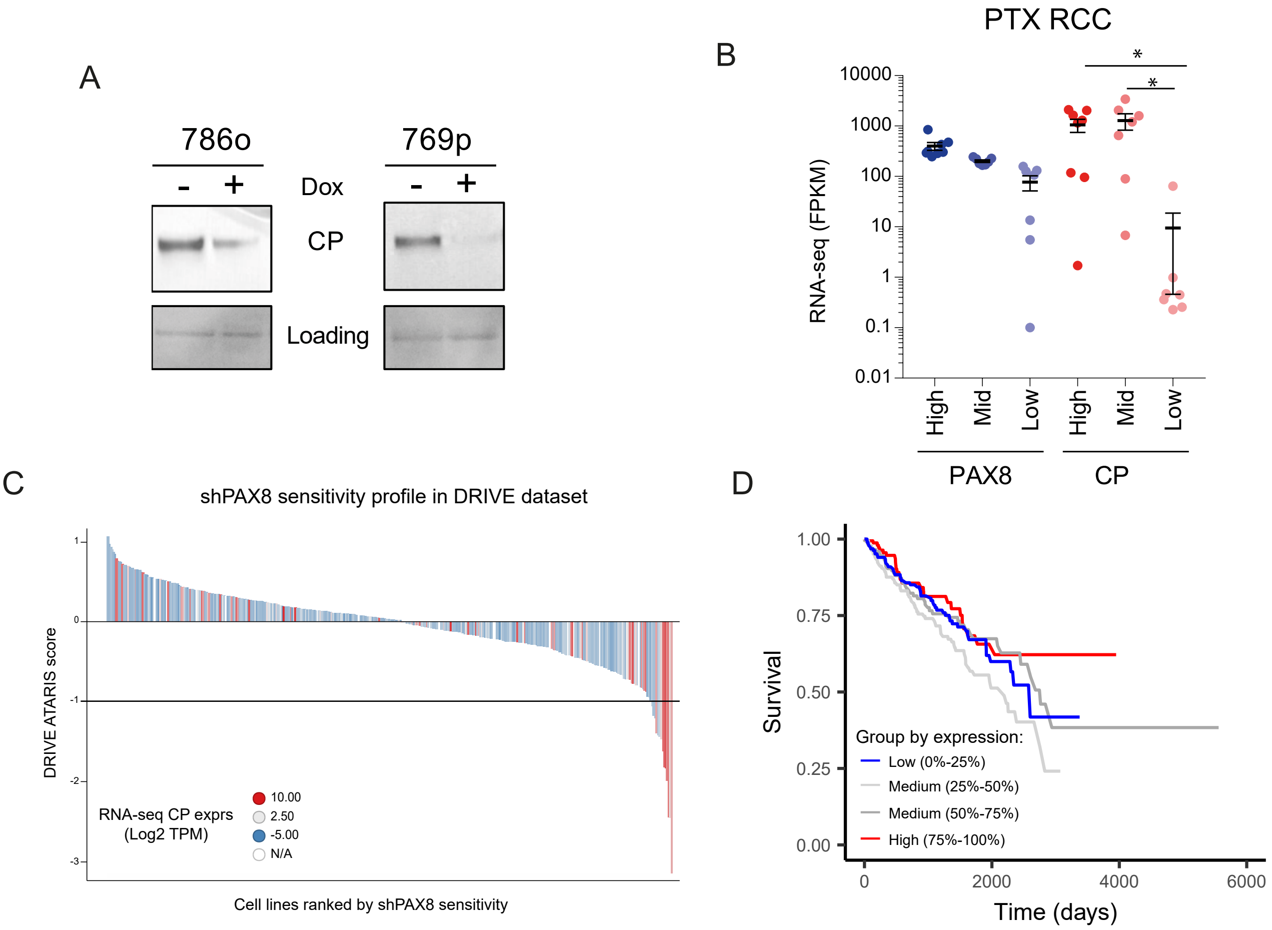

Supplementary Figure 4. CP expression tracks with PAX8 expression and sensitivity

A) Western blot analysis of conditioned media from 786o and 769p cells treated for 4 days with doxycycline to activate shPAX8. Unspecific band is used as a loading control. B) RNA-seq expression of PAX8 and CP across the Novartis Primary Tumor Xenografts collection segregated evenly according to PAX8 High, Mid and Low expression. * = p<0.005 based on Welch's T-test. C) Waterfall plot for the sensitivity of cell lines to PAX8 knockdown as reported in the DRIVE dataset. Each histogram bar is a cell line and bars are color-coded based on the CP expression level (by RNA-seq). Horizontal line at -1 defines treshold for significant sensitivity. D) Kaplan-Meyer plot representing the survival of TCGA RCC cases segregated based on PAX8 expression (4 equal-sized bins).

Original western blot images

Figure 1D

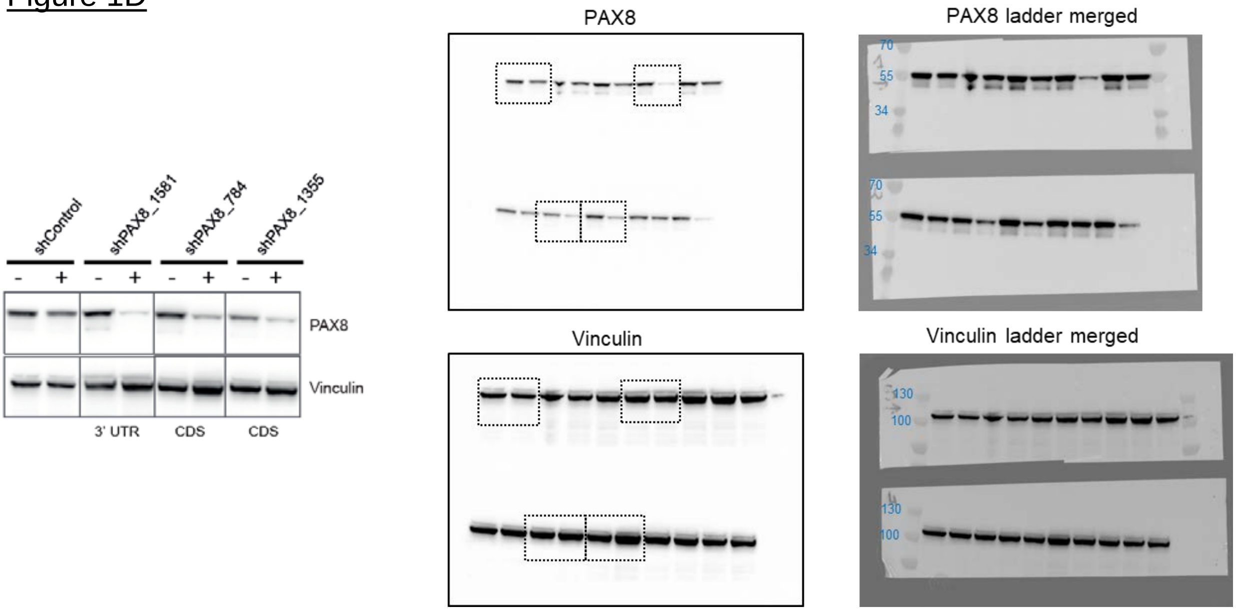

Figure 3C

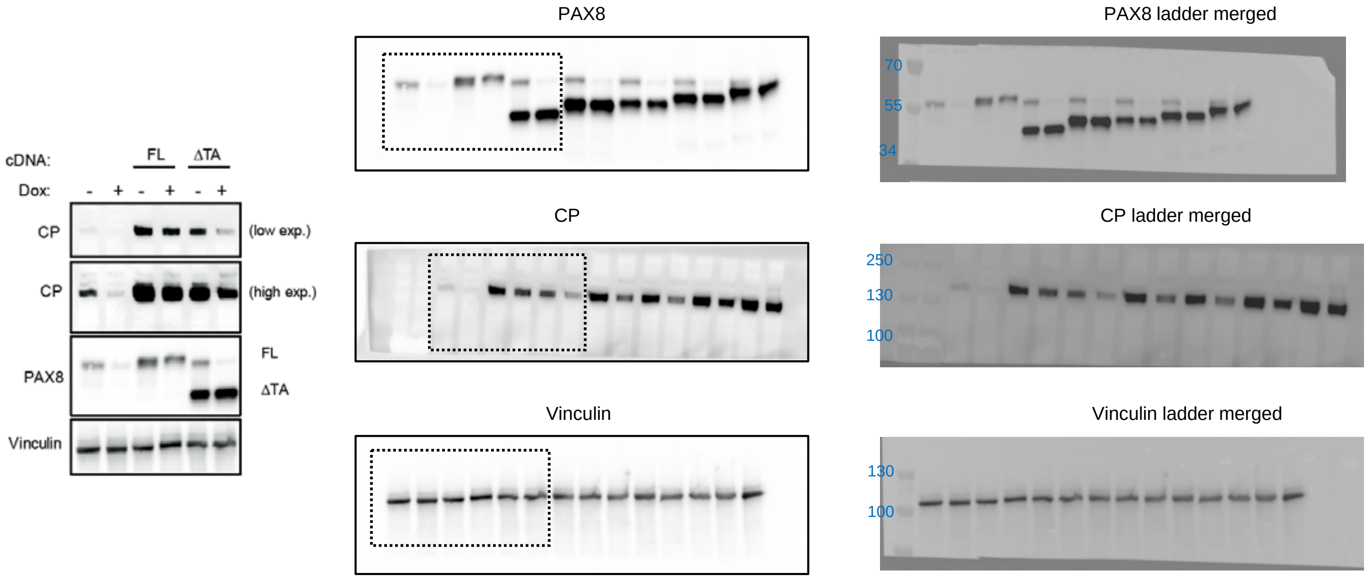

Supplement: Supplementary file 1 — Supplementary Information [file 41467_2019_11672_MOESM1_ESM.pdf]
