# Supplementary material for: PAX8 activates metabolic genes via enhancer elements in Renal Cell Carcinoma
Source: Nat Commun. 2019 Aug 20;10:3739. doi: 10.1038/s41467-019-11672-1 (PMC6702156; doi:10.1038/s41467-019-11672-1)
Supplement: Supplementary file 2 — Description of Additional Supplementary Files [file 41467_2019_11672_MOESM2_ESM.pdf]

Description of Additional Supplementary Files

File Name: Supplementary Data 1
Description: Differentially expressed genes in 4 RCC lines upon shPAX8. Log2FC and Adjpvalue are calculated based on One-way Anova and corrected for multiple testing
