# Supplementary material for: PAX8 activates metabolic genes via enhancer elements in Renal Cell Carcinoma
Source: Nat Commun. 2019 Aug 20;10:3739. doi: 10.1038/s41467-019-11672-1 (PMC6702156; doi:10.1038/s41467-019-11672-1)
Supplement: Supplementary file 3 — Supplementary Data 1 [file 41467_2019_11672_MOESM3_ESM.pdf]

Supplementary Data 1

Gene_symbol	log2FoldChange	Adj.pvalue
ALDH3B1	1.288805032	2.70E-160
UBE2V2	-1.360706772	9.80E-108
RPA3	-1.816459127	1.10E-106
METAP1	-1.670784622	6.20E-103
CP	-1.71601299	1.30E-92
ATP2B4	1.922504125	4.00E-86
PCDH7	2.900734446	1.30E-84
SMPD1	1.499942434	1.30E-82
SMC2	-0.965688892	5.70E-76
EI24	-1.717512825	2.40E-72
CPA4	3.48729031	1.70E-70
PRDM1	3.410670593	2.00E-66
MAT2B	-1.504363392	1.60E-65
GBP2	1.613546438	2.80E-62
HMGB1	-0.991865836	1.20E-60
TMEM97	-1.269593272	1.20E-55
MCTP1	-1.164857915	1.20E-54
ADAM19	1.521452269	2.20E-54
HDGF	-0.809737942	1.90E-53
GPR180	-1.335652361	2.80E-53
MAD2L1	-1.51247515	3.20E-52
SPOCD1	2.416377212	3.70E-50
HIST1H3A	-1.311825036	1.00E-48
P3H4	1.583338865	7.20E-48
TOM1L2	1.164973754	1.30E-47
LPCAT2	1.035096223	1.40E-46
KAZN	-1.0751906	2.90E-46
HDAC5	1.692568076	5.10E-46
GAS2L3	-1.144659556	7.70E-46
GNPNAT1	-1.061159574	9.00E-45
BMF	2.138282907	1.40E-43
KHDRBS3	-1.160734314	3.30E-43
POLD4	1.078760813	1.40E-42
SSX2IP	-1.086012186	2.60E-42
CLU	1.879343805	3.10E-42
LURAP1L	2.095003076	8.30E-42
SUMF1	-1.428127588	9.50E-42
FAM214B	1.36617429	1.60E-41
TFDP1	-0.741277405	5.20E-41
SEZ6L2	1.361880766	9.80E-41
ITGA5	1.160316102	2.00E-40
TGFBR2	1.113545626	5.50E-40
RBFOX2	0.694418499	7.30E-40
SFXN3	0.703256048	1.50E-39
ESCO2	-1.153448567	2.00E-39
LPXN	-1.291910036	3.50E-39
ANKS6	-0.940476299	2.20E-38
GSN	1.427840552	9.10E-38
THOP1	-1.371812577	1.30E-37

DDAH1	-1.42940679	1.70E-37
H2AFZ	-0.842014538	3.20E-37
ROBO1	0.988869487	4.30E-37
CAPN5	1.822202506	7.40E-37
TFAM	-0.942513523	1.80E-36
HAUS6	-0.792589874	2.10E-36
FAR1	-1.156923934	2.20E-36
FAM171A1	-1.521394767	4.10E-36
HIST1H2BC	-0.861779355	1.30E-35
RASA4	1.457056007	1.50E-35
GPRIN1	-0.930269631	2.00E-35
PTMA	-1.206886165	2.30E-35
MCM2	-0.976500676	1.30E-34
UBE2D3	-0.702847219	3.00E-34
C1QTNF6	1.507640018	6.30E-34
HIST1H4D	-1.317982897	8.70E-33
MEF2D	0.874993036	7.40E-32
TMEM14A	-1.02535763	7.90E-32
RNF138	-0.962900934	8.10E-31
POLA1	-0.897306125	8.50E-31
CHAF1B	-0.744156742	9.50E-31
GMNN	-0.917186722	1.10E-30
ORAI3	1.137739666	1.50E-30
MRPL11	-0.963004276	1.90E-30
RNF144B	-1.362055844	2.90E-30
HIST1H2AB	-1.17691387	3.70E-30
KIAA0101	-1.062337385	4.00E-30
LAMB2	1.234841812	9.90E-30
COQ10B	0.666139764	1.10E-29
TBL1XR1	-0.940252953	1.80E-29
CALB2	2.298256509	2.50E-29
CX3CL1	1.080815299	7.40E-29
BRI3BP	-0.985032209	1.30E-28
SSBP2	1.033177126	1.40E-28
BMP1	1.304989316	1.90E-28
PAX8	-1.473591199	2.10E-28
RP1-34B20.21	-1.129435119	4.00E-28
CIDEC	2.621117859	5.10E-28
VGLL4	0.720799792	9.70E-28
GRPEL2	-0.717033271	1.20E-27
HNRNPLL	1.017664088	1.20E-27
HIST1H2BD	-0.941185552	1.40E-27
NEMP1	-0.884383675	1.80E-27
DST	1.150740572	3.60E-27
RABEP1	-0.810196878	7.20E-27
SAE1	-0.695650968	7.50E-27
AKR1C1	2.145228053	8.50E-27
WDR76	-1.017491069	1.10E-26
FIGNL1	-1.067895864	1.70E-26
SHMT1	-0.889601216	1.70E-26
CD3EAP	-1.056108587	2.10E-26
CA2	-1.428149686	2.90E-26
SLCO2B1	1.661110802	3.70E-26
SPC25	-1.128972282	4.00E-26
ANAPC1	-0.71932613	4.50E-26

SEMA3B	2.066200879	4.50E-26
MSH2	-0.680227526	5.50E-26
GINS2	-1.172437506	5.70E-26
CREB5	-1.004148235	8.30E-26
MMS22L	-0.789571662	8.40E-26
EFHB	-1.817885223	8.70E-26
IFITM10	2.060162017	9.30E-26
PFAS	-0.930914044	9.70E-26
OAS3	-1.122399992	1.10E-25
TYMS	-1.05685565	1.10E-25
PCDH20	-2.092893457	1.60E-25
ZNF467	2.083574534	2.10E-25
GTPBP3	-0.948961753	2.40E-25
MCM10	-1.24392899	2.70E-25
PBX2	0.773258918	2.80E-25
POLD3	-0.940421126	3.10E-25
LAMA5	1.241642286	4.20E-25
HIST1H1D	-1.437073495	5.60E-25
IL7R	1.702039903	9.40E-25
GPR160	-1.162013901	1.10E-24
TCP11L2	1.417004219	1.90E-24
G0S2	1.891095764	3.00E-24
RBL1	-0.888539453	3.70E-24
TPRG1L	0.851434129	3.80E-24
HIST1H4L	-1.688537584	4.10E-24
IDS	0.905470176	5.30E-24
COL6A1	1.205697439	5.40E-24
NCAPG	-0.979320303	1.30E-23
TRIM2	1.314187602	1.40E-23
FANCI	-1.131195514	1.50E-23
HSPB8	0.869357197	1.50E-23
HIST1H2AD	-1.107929506	1.60E-23
MRE11A	-0.8373522	1.90E-23
HIST1H4C	-0.877121215	3.60E-23
WDHD1	-0.861717848	4.20E-23
SLFN5	1.440729591	4.50E-23
USP1	-0.70332066	6.40E-23
MFI2	0.906904156	9.10E-23
PGAP3	0.865111667	9.90E-23
HIST2H2AC	-0.926102055	1.10E-22
RRM2	-0.904556727	1.50E-22
DCP2	-0.933215035	2.00E-22
TTF2	-0.719481344	2.10E-22
BIRC5	-1.231425051	3.20E-22
NUSAP1	-1.063150224	3.30E-22
MCM7	-1.099418641	4.20E-22
SH3BGRL3	0.727873799	7.50E-22
ATAD2	-1.022606359	8.20E-22
HIST2H2AB	-0.917005776	1.10E-21
PRPF38A	-0.801604523	1.60E-21
GUCA1C	-2.890927021	2.00E-21
SMC4	-0.932382779	2.30E-21
SEPW1	1.013831735	3.10E-21
C18orf54	-1.299549571	3.70E-21
NAV1	1.083236136	3.70E-21

CYLD	0.705669441	5.20E-21
MCM3	-0.813192342	7.20E-21
DBF4	-0.921189742	9.30E-21
DIAPH3	-1.375136329	9.80E-21
FAM234A	0.698121521	1.20E-20
CERCAM	1.043618595	1.30E-20
SMIM14	1.346816695	1.40E-20
MRS2	-0.853662458	1.70E-20
PROS1	0.77982832	1.70E-20
HIST1H2BJ	-1.216283731	2.20E-20
RPN1	-0.747043432	2.20E-20
RAB8A	-0.724433297	2.40E-20
CHPF	1.152433479	2.50E-20
MTBP	-0.857587205	2.70E-20
ORAI2	0.663271623	2.70E-20
THOC7	-1.072035386	3.90E-20
CTSA	0.862968603	4.00E-20
DNAJC9	-0.952513963	4.90E-20
HIST1H2AL	-1.286422461	5.20E-20
SMYD2	-0.728263197	6.00E-20
HIST1H3F	-1.356186596	6.30E-20
RHOU	1.317015373	6.60E-20
DTD1	-0.795177515	7.50E-20
INIP	-0.74921063	1.30E-19
HIST1H2BE	-1.223876222	1.40E-19
ARNTL	0.989093468	1.50E-19
KIFC1	-1.068734503	1.60E-19
PCNA	-0.803861306	2.10E-19
HIST2H4A	-1.154331856	2.70E-19
RNASEH2A	-0.819292701	2.70E-19
PLOD1	0.720467999	2.70E-19
FAM214A	1.132063806	2.70E-19
HIST1H2AM	-1.113484403	3.20E-19
CACYBP	-0.599920022	3.30E-19
ZNF862	1.127434844	3.40E-19
TMPO	-0.996158196	3.70E-19
KLHL28	0.799316558	3.70E-19
ZMIZ1	1.100581312	4.00E-19
NCAPD3	-0.796720426	5.20E-19
CSE1L	-0.752745691	5.40E-19
NIPAL3	1.077212866	5.80E-19
RANBP1	-0.755293564	6.10E-19
NEMP2	-0.958272287	7.30E-19
ELOVL7	-1.035851167	7.40E-19
AMPD3	1.252763987	7.40E-19
BLM	-1.068554422	7.70E-19
HIST2H3D	-1.343721	8.20E-19
GLO1	-0.70938103	8.20E-19
HIST1H2AH	-1.249304562	9.50E-19
RCC1	-0.871948183	9.60E-19
GPSM2	-0.785768644	1.10E-18
DUSP13	2.727982606	1.10E-18
CENPU	-1.112558474	1.20E-18
C1orf186	-0.959364637	1.20E-18
NANOS1	1.419128275	1.30E-18

DCBLD1	1.341786338	1.40E-18
SCG5	2.028257124	1.60E-18
DACT1	2.083126589	1.80E-18
HIST1H2BG	-1.180395609	1.90E-18
EVA1B	0.956871389	2.30E-18
DCLRE1A	-0.772038165	2.50E-18
SREK1IP1	-0.769164763	2.50E-18
DSCC1	-0.928003154	2.80E-18
CNTNAP3	1.505085986	3.20E-18
HIST1H2BK	-0.869719566	3.40E-18
HIST1H3J	-1.476849149	3.50E-18
HIST1H2AJ	-1.216963205	3.50E-18
ADGRF4	1.621272911	3.70E-18
CLN8	0.677172573	3.80E-18
UNG	-0.727321507	5.60E-18
MCM4	-0.869830802	6.00E-18
NOB1	-0.642971189	6.20E-18
GATSL2	0.971301934	6.20E-18
FAM111B	-1.11738203	6.80E-18
CENPI	-1.011242017	7.10E-18
HIST2H2BE	-0.966175549	1.20E-17
G3BP1	-0.890376482	1.20E-17
KIAA1147	-0.683917443	1.20E-17
GPC1	0.929007506	1.20E-17
CHAF1A	-0.785336658	1.30E-17
HSPG2	0.839002818	1.50E-17
HIST1H4B	-0.836610191	1.60E-17
MRPL3	-0.617734202	1.70E-17
TMEM178B	-1.28095708	1.90E-17
MCM5	-0.774536653	1.90E-17
HIST2H2BF	-1.173416212	2.00E-17
RFC3	-0.868657118	2.00E-17
BLCAP	0.622046809	2.30E-17
MOSPD1	0.830937591	2.30E-17
HIST1H1B	-1.250288746	2.40E-17
MTFR1	-0.642215236	2.70E-17
HMGB3	-0.63031227	2.70E-17
S100A4	1.739113231	2.70E-17
RNF128	0.735757108	2.80E-17
KATNAL1	-1.02397931	3.30E-17
BTBD11	0.945684859	3.40E-17
SAAL1	-0.819053584	3.80E-17
MLPH	1.112325765	4.10E-17
CLEC4E	-1.984753703	4.20E-17
HIST1H2BB	-1.357010287	4.20E-17
MAPRE3	1.165162142	4.60E-17
HIST1H2BO	-1.207666108	4.80E-17
ECT2	-0.860259654	5.10E-17
CENPQ	-1.03360922	6.10E-17
RAD51AP1	-1.157635913	6.30E-17
LRRN4	-1.594872168	8.00E-17
EXO1	-1.077233001	8.40E-17
TIPIN	-0.819237592	8.70E-17
ADAMTS3	-1.232152891	8.80E-17
TAF4B	-0.984781643	8.80E-17

GFPT2	2.227100243	9.30E-17
PARP1	-0.641384121	1.10E-16
COL6A2	0.885294784	1.10E-16
HIST1H2BN	-0.786367923	1.20E-16
SIPA1L3	-0.760889242	1.20E-16
HPGD	2.112576016	1.30E-16
CASP3	-0.789867411	1.40E-16
CDK2	-0.797926291	1.50E-16
HIST1H3C	-1.104269353	1.60E-16
BRWD3	0.739931063	1.60E-16
MMP24-AS1	1.012741944	1.90E-16
BFSP1	1.716373426	1.90E-16
E2F1	-0.990214583	2.00E-16
INSIG1	-0.912402052	2.00E-16
RHBDF1	0.724823661	2.30E-16
HELLS	-0.936666853	2.50E-16
GGH	-0.669578304	2.50E-16
FAM72D	-1.279190698	2.80E-16
PLSCR4	1.726402078	3.00E-16
PKMYT1	-1.170633974	3.50E-16
SET	-0.511388928	3.60E-16
THRB	-1.008442567	4.10E-16
PRIM1	-1.107950824	4.50E-16
DCDC2	-1.1704573	4.90E-16
SH3BP5	0.869502676	6.00E-16
CD68	1.107667091	6.10E-16
TPK1	-1.240572893	6.20E-16
DEK	-0.705283323	7.60E-16
FANCA	-0.926030281	7.90E-16
DIRAS2	2.071056894	8.50E-16
HIST1H2AE	-1.05765035	9.30E-16
RAB13	0.737691871	1.00E-15
NNMT	-1.016542371	1.10E-15
GAREM	0.867393761	1.10E-15
PM20D2	-0.875684408	1.20E-15
PDE12	-0.829846284	1.20E-15
HIST1H2BF	-1.04053329	1.30E-15
DSG2	-0.945327164	1.30E-15
PTTG1	-0.757030645	1.30E-15
SUV39H2	-0.704777351	1.30E-15
TMEM59	0.883551421	1.30E-15
HIST1H4A	-0.753586365	1.50E-15
CDKN1A	1.119582816	1.80E-15
TNFRSF1A	0.615287007	1.90E-15
ABCA7	1.337359746	1.90E-15
KIF4A	-1.010257211	2.00E-15
TSC22D1	0.814261326	2.00E-15
AKR1C3	1.756882035	2.20E-15
SIDT2	0.943641623	2.30E-15
COL4A2	-1.235413903	2.60E-15
HIST1H2AG	-1.0285196	3.40E-15
BUB1	-0.907007607	3.40E-15
TMEM185B	-0.735411458	3.60E-15
GXYLT2	1.240772612	3.60E-15
CTIF	1.268636945	3.60E-15

EVI5L	1.290343694	3.60E-15
ATAD5	-0.755993014	3.70E-15
CLSPN	-1.056809333	4.00E-15
NCAPD2	-0.688356491	4.30E-15
CDCA7	-0.983801354	4.40E-15
IL11	1.981583895	5.20E-15
DEPDC1B	-1.12841227	5.30E-15
THRA	1.034421464	5.70E-15
RMI2	-1.021740756	6.20E-15
NCAPG2	-0.776625613	6.80E-15
HIST1H3B	-1.105386879	7.20E-15
CDC45	-0.976471427	7.60E-15
LLGL2	1.164520723	7.60E-15
QSOX1	0.749704086	7.70E-15
CWC27	-0.641436105	7.80E-15
U2SURP	-0.536689568	8.40E-15
SLC22A4	1.220030387	8.90E-15
SRPX2	1.986816243	9.20E-15
RBMX	-0.545935266	1.00E-14
HIST1H2BL	-1.145025206	1.10E-14
HIST1H3I	-1.294015198	1.20E-14
FAM111A	-0.770029468	1.70E-14
PNRC1	0.979812739	1.70E-14
ANP32E	-0.706993933	1.90E-14
HES1	0.918629796	1.90E-14
DUT	-0.645313821	2.10E-14
SYNJ2	0.993207311	2.10E-14
TNFRSF21	1.191762324	2.10E-14
KIF15	-1.033695963	2.20E-14
NELFCD	-0.742551121	2.60E-14
FAM3C	1.02741124	2.80E-14
CEP126	0.979079332	2.90E-14
SEC14L2	0.751156684	3.10E-14
EPHX1	0.777255976	3.30E-14
PPL	1.379823929	3.30E-14
MN1	1.578981434	3.30E-14
NKIRAS2	0.639904278	3.80E-14
FKBP5	-1.045166325	4.00E-14
PLXND1	0.826055936	4.10E-14
SP4	-0.85765063	4.20E-14
CENPW	-1.077782567	5.10E-14
CDCA5	-0.945472808	5.50E-14
SYTL3	-1.073352908	5.70E-14
PITPNM2	0.925849845	5.80E-14
ZCCHC24	1.053032941	5.80E-14
GOLT1A	1.489887294	6.00E-14
PPP1R9A	-0.685211409	6.10E-14
MANBA	0.986530602	6.80E-14
KRT81	2.452931218	7.50E-14
CACFD1	0.892206518	8.00E-14
TNPO3	-0.684981672	9.10E-14
MACROD2	-1.150600219	9.60E-14
SSB	-1.029976287	9.90E-14
RAB7B	1.599592873	9.90E-14
KCMF1	-0.634596079	1.00E-13

KLHL23	-0.62775173	1.10E-13
MSR1	1.441281476	1.10E-13
ABCF1	-0.629392373	1.30E-13
LYST	0.876145312	1.60E-13
UMPS	-0.58964327	1.70E-13
PLD3	0.769983107	1.70E-13
ABCC3	1.136170045	1.70E-13
STAT1	-1.262144595	2.10E-13
OSTM1	-0.880866364	2.10E-13
SEC24B	-0.630060053	2.10E-13
DDR1	1.364789015	2.10E-13
PPP1R14C	-1.257490029	2.20E-13
PCDHGB7	1.44645087	2.30E-13
ETFB	0.670714944	2.40E-13
EDEM3	0.542519513	2.70E-13
RPE	-0.587640337	2.80E-13
STIL	-0.900984462	2.90E-13
CCNE2	-1.071207306	3.00E-13
MCM8	-0.686664539	3.00E-13
PRSS23	1.504188105	3.00E-13
ADGRB2	1.681775418	3.00E-13
CENPA	-0.989178023	3.20E-13
PINK1	0.874279794	3.20E-13
KIF20B	-0.793294902	4.00E-13
ZWINT	-0.937814766	4.30E-13
SFXN2	-0.809165059	4.50E-13
ST3GAL5	1.443605962	4.60E-13
H2AFX	-0.82052803	4.70E-13
ADAM12	1.285985845	5.80E-13
CENPH	-0.853974266	5.90E-13
PKDCC	0.924621497	5.90E-13
NEFL	-1.06182384	6.50E-13
ENDOD1	1.063966869	6.80E-13
TNC	2.04580145	7.20E-13
E2F8	-1.331204098	7.60E-13
WFDC3	1.286474512	8.50E-13
CSRP2	-1.064285481	1.00E-12
FANCD2	-0.874152486	1.00E-12
KIAA1524	-0.849983991	1.00E-12
H2BFS	-0.732498549	1.00E-12
TICRR	-0.722251485	1.20E-12
SPAG7	0.654414064	1.20E-12
SAPCD2	-0.848715089	1.30E-12
CEP78	-0.614353399	1.30E-12
RPA1	-0.612722307	1.30E-12
EBNA1BP2	-0.605518148	1.30E-12
GRIP1	0.887795335	1.40E-12
CDK1	-0.947158491	1.50E-12
CLOCK	-0.668144952	1.50E-12
GAN	-0.641838496	1.70E-12
IRGQ	0.556292374	1.70E-12
AP3S2	0.58643899	1.70E-12
CRAT	0.748131485	1.70E-12
HIST1H3D	-0.988562982	1.80E-12
TGIF1	-0.668220143	1.80E-12

SLC47A2	1.970822267	2.00E-12
CSTF2	-0.581941118	2.30E-12
CTSD	0.77296868	2.40E-12
SKA3	-0.96309783	2.60E-12
ADAMTS10	1.868257319	2.60E-12
DNA2	-0.743363767	2.90E-12
MMGT1	-0.858158466	3.00E-12
TCN2	1.219989853	3.50E-12
FUBP1	-0.64837773	3.60E-12
CDC23	-0.576679457	4.10E-12
PBX1	0.988021414	4.10E-12
LIPH	1.404703228	4.10E-12
FAM149A	-0.976846967	4.20E-12
RAPGEF2	0.578921016	4.30E-12
ZMAT3	0.83303517	4.70E-12
DHFR	-1.004386519	5.10E-12
MSRB2	0.764640335	5.20E-12
GATS	0.973056998	5.60E-12
ADAP2	-0.840587711	5.80E-12
CYB561D1	0.746950393	6.90E-12
ANTXR2	0.868665565	7.20E-12
HIST1H1E	-0.931168677	7.50E-12
KIF14	-0.855124022	7.60E-12
SPC24	-0.66743113	8.00E-12
HIST2H3A	-1.089496333	8.40E-12
HIST2H3C	-1.089496333	8.40E-12
BRCA1	-0.761790911	9.20E-12
CENPF	-0.860115178	9.30E-12
SERP1	0.542888022	9.40E-12
AGPAT3	-0.670270031	9.60E-12
TK1	-0.857689603	1.10E-11
UBE2T	-0.808211583	1.10E-11
PTPN3	-0.802292634	1.10E-11
HNF1B	-1.010991595	1.20E-11
SPARC	1.475231321	1.20E-11
UHRF1	-0.89711204	1.40E-11
DNAJB9	0.995813711	1.40E-11
N4BP3	-1.669484177	1.50E-11
